# Technological Innovations and Research Frontiers in Interventional Therapy for Mitral Regurgitation 

**DOI:** 10.31083/RCM46175

**Published:** 2026-05-29

**Authors:** Shuang Wang, Aili Wang, Yuna Huang, Jinping Liu, Bin Wang

**Affiliations:** ^1^Department of Cardiovascular Ultrasound, Zhongnan Hospital of Wuhan University, Wuhan University, 430071 Wuhan, Hubei, China; ^2^Department of Cardiovascular Surgery, Zhongnan Hospital of Wuhan University, 430071 Wuhan, Hubei, China

**Keywords:** mitral regurgitation, transcatheter intervention, structural heart disease, medical devices

## Abstract

Mitral regurgitation (MR) is a common valvular heart diseasewhose prevalence continues to increase with population aging, posing a serious threat to human health in the advanced stages of the disease. Sole reliance on medication and traditional surgical treatments can no longer meet the therapeutic needs of all patients. Transcatheter interventional therapy is gradually emerging as a promising new treatment option. Recently, technologies for the transcatheter interventional treatment of MR have advanced rapidly, with expanding indications and a continuous stream of new devices. The field has entered a phase of accelerated development in the treatment of structural heart disease, demonstrating broad clinical prospects. This article reviews the key technologies and developmental trends in the current transcatheter interventional treatments for MR, aiming to provide a theoretical basis and rationale for the safe and standardized implementation and broader adoption of these technologies.

## 1. Introduction

Mitral regurgitation (MR) is a clinically common valvular heart disease that poses a serious threat to human health in its advanced stages. According to statistics [1], the prevalence of MR reaches 9.3% among individuals aged 75 and older in the United States, while hospital-based data in Europe report an MR prevalence of 24.4%, with moderate to severe MR accounting for 5.2%. The prevalence of MR increases significantly with age, and it is estimated that approximately 7.5 million patients in China require interventional treatment for MR [2]. Over two-thirds of patients are ineligible for surgical treatment due to high-risk factors such as advanced age and comorbidities, with a five-year mortality rate as high as 50% [3].

With the rising number of MR cases, pharmacological therapies have proven ineffective in altering disease progression, while traditional surgical repair or replacement cannot be performed on all patients because of underlying comorbidities [4,5]. Transcatheter mitral valve interventions (TMVI) have seen rapidly growing demand due to their advantages, including lower risk, minimal invasiveness, and fewer postoperative complications [6,7,8]. A comparison of various techniques is presented in Table 1. With technological advancements, the indications for TMVI continue to expand, and innovative devices are constantly emerging [9,10,11,12,13]. TMVI has now become a highly promising procedure in the field of structural heart disease [14,15,16,17,18]. This review provides a comprehensive review of the key technologies and recent advances in TMVI for MR, aiming to promote the safe and standardized application of these techniques and to offer a theoretical foundation and evidence for their role in clinical practice.

**Table 1.** **Comparison summary of TEER, TMVR, and annuloplasty**.

Technology	TEER	TMVR	Annuloplasty
Indications	1. Severe primary degenerative mitral regurgitation with inadequate response to medical therapy; 2. Severe secondary functional mitral regurgitation with persistent heart failure symptoms despite optimal medical treatment, accompanied by EROA >0.3 cm^2^ and LVEF 30%–50%; 3. Patients with moderate to severe MR who are at high risk for or contraindicated to surgical intervention (e.g., advanced age, multiple organ comorbidities); 4. Central regurgitation, mitral valve (MV) area >4 cm^2^, without significant mitral stenosis or severe annular calcification.	1. Moderate to severe MR with complex anatomical structures, for which TEER is ineffective or inappropriate (e.g., leaflet length <7 mm, large flail gap/width, multi-leaflet segmental prolapse); 2. Mixed MV disease with MV area <3.5 cm^2^; 3. Patients at high risk for or contraindicated to surgery, without high risk of left ventricular outflow tract obstruction (LVOTO); 4. MV lesions with thin, short, fragile, or calcified leaflets.	1. Moderate to severe regurgitation caused by mitral annular dilatation (especially functional regurgitation); 2. Primary mitral regurgitation (PMR) combined with annular dilatation requiring simultaneous correction of annular morphology; 3. Suitable for high-risk surgical patients with annular dilatation-type regurgitation.
Anatomical limitations	1. Patients with insufficient leaflet length or low leaflet coaptation height have a high risk of residual regurgitation postoperatively; 2. Efficacy is limited in patients with significant anterolateral annular dilatation or excessive left atrial enlargement; 3. Technical difficulty is increased in cases of severe MAC or excessively wide regurgitant orifice (e.g., >19 mm); 4. Not applicable for mitral stenosis (MV area <2 cm^2^).	1. Abnormal annular size/morphology (e.g., irregular annulus) and severe mitral annular calcification (MAC) increase delivery difficulty; 2. Patients with left ventricular outflow tract stenosis or anatomical abnormalities have a high risk of postoperative LVOTO; 3. Poor tolerance in patients with significantly impaired right ventricular function or severe pulmonary hypertension; 4. The transfemoral approach is limited by vascular conditions and interatrial septal anatomy.	1. Isolated annuloplasty yields poor outcomes in cases of severe leaflet lesions (e.g., large-area leaflet prolapse, perforation); 2. Severe annular calcification poses challenges for annuloplasty ring fixation and stability; 3. Simple annuloplasty is difficult to reverse cardiac insufficiency in patients with severe left ventricular enlargement and poor cardiac function (LVEF <30%); 4. Interventional annuloplasty is limited by annular anatomical morphology and surrounding tissue obstruction.
Key Efficacy indicators	1. Degree of residual regurgitation postoperatively (target: ≤mild regurgitation); 2. 6-minute walk distance and KCCQ score (assessing quality of life improvement); 3. Heart failure hospitalization (HFH) rate and all-cause mortality; 4. Transvalvular pressure gradient (avoiding postoperative mitral stenosis); 5. Reduction in left atrial/left ventricular volume (assessing reverse remodeling).	1. Regurgitation elimination rate (target: complete elimination or ≤mild residual regurgitation); 2. Prosthetic valve function (transvalvular pressure gradient, paravalvular leak incidence); 3. 2-year survival rate and heart failure rehospitalization rate; 4. Improvement in left ventricular ejection fraction (LVEF); 5. Valve thrombosis incidence.	1. Degree of regurgitation control postoperatively (target: ≤mild regurgitation); 2. Annular diameter reduction rate and stability (avoiding postoperative annular redevelopment); 3. Improvement in NYHA cardiac function classification; 4. Long-term survival rate and incidence of major adverse cardiovascular events; 5. Reduction in left ventricular end-diastolic volume index.
Common technical challenges	1. A single clip is insufficient to fully control regurgitation in patients with a wide regurgitant orifice (>19 mm), and the technical difficulty increases when multiple clips are required; 2. Precise leaflet capture under transesophageal echocardiography guidance, especially in patients with poor leaflet mobility; 3. Maintenance of vital signs during surgery in elderly patients or those with multiple organ dysfunction; 4. Prevention of complications such as single-leaflet clip dislodgement and leaflet injury.	1. Precise transcatheter delivery and positioning of the prosthetic valve (especially overcoming multi-curved anatomy in the transfemoral approach); 2. Avoidance of intraoperative injury to subvalvular structures (chordae tendineae, papillary muscles) and left ventricular outflow tract; 3. Immediate management of postoperative paravalvular leaks; 4. Prevention of access-related complications such as bleeding and infection in the transapical approach.	1. Surgical procedure: Cardiopulmonary bypass-related complications (e.g., renal injury, infection), as well as precise matching and fixation of the annuloplasty ring; 2. Interventional procedure: Positioning and anchoring of the delivery system after crossing the interatrial septum, avoiding injury to surrounding valves/vessels; 3. Balancing the degree of annular constriction to prevent mitral stenosis due to excessive narrowing; 4. Maintenance of intraoperative hemodynamic stability in patients with concurrent left ventricular dysfunction.
Completed or ongoing clinical trials	EVEREST I/II, COAPT, MITRA-FR, RESHAPE-HF, EXPAND, CLASP I/II, CLASP HF, etc.	ENCIRCLE Trial, APOLLO Trial, SUMMIT-MAC Study, ATLAS Trial, Mi-thos® Clinical Trial, etc.	REDUCE-FMR, TRISCEND, RESILIENT, etc.

TEER, transcatheter edge-to-edge repair; TMVR, transcatheter mitral valve replacement; KCCQ, Kansas City Cardiomyopathy Questionnaire; NYHA, New York Heart Association; EVEREST, Endovascular Valve Edge-to-Edge Repair Study; COAPT, cardiovascular outcomes assessment of the MitraClip percutaneous therapy; MR, mitral regurgitation.

## 2. Transcatheter Mitral Valve Repair

**Fig. 1.**
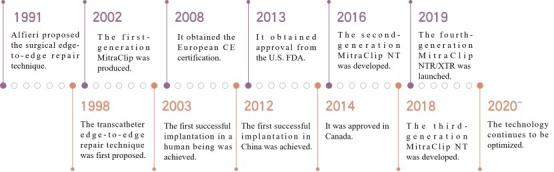
**The historical evolution process of MitraClip**.

Interventional techniques for MR include two major categories: transcatheter mitral valve repair (TMVr) and transcatheter mitral valve replacement (TMVR) [19,20,21,22,23]. The studies and instruments involved in this article are shown in the graphical abstract. TMVr is further subdivided into transcatheter edge-to-edge repair (TEER), annuloplasty, and artificial chordae repair. Currently, a total of 6 TMVr products have obtained the European Conformity (CE) certification internationally. Among them, TEER products are the most mature, represented by MitraClip and PASCAL, both of which have received CE certification. MitraClip is the only product approved by three authorities: the US Food and Drug Administration (FDA), the China National Medical Products Administration (NMPA), and CE. It is also the most evidence-based, widely used, and mature technology. The historical evolution process of MitraClip is shown in Fig. 1. In China, two domestic devices, ValveClamp and DragonFly, were approved for marketing by NMPA in 2023.

### 2.1 TEER Leads in Mitral Valve Interventional Therapy, With Product Iteration Expanding Patient Population and Optimizing Clinical Efficacy

#### 2.1.1 MitraClip Achieves Favorable Safety and Efficacy in Real-World Cohort Studies

Since the first human implantation of MitraClip in 2003, a large number of clinical studies on TEER have been conducted over the past two decades, continuously verifying its clinical efficacy and expanding patient indications. The Endovascular Valve Edge-to-Edge Repair Study (EVEREST) I [24] and EVEREST II [25] trials have laid the foundation for its clinical application. The EVEREST II trial was a multicenter, randomized study that allocated 279 patients with severe (3+ or 4+) MR to the MitraClip percutaneous repair group (n = 178) or the conventional mitral valve (MV) surgery group (n = 80) at a 2:1 ratio, with a 5-year follow-up. The results demonstrated that the surgery group achieved superior outcomes at 5 years in terms of the composite endpoint of "no death, no MV surgery/reoperation, and no severe (3+ or 4+) MR" (64.3% vs. 44.2%, *p* = 0.01), reduction in severe MR (1.8% vs. 12.3%, *p* = 0.02), and lower rates of surgery/reoperation (8.9% vs. 27.9%, *p* = 0.003). However, there was no significant difference in 5-year mortality between the two groups (26.8% vs. 20.8%, *p* = 0.4). Additionally, the incidence of MV dysfunction requiring surgery during the 1–5 year follow-up period was low in both groups (76.2% vs. 77.7%; *p* = 0.77), confirming that both repair techniques exhibit durability in MR reduction and favorable long-term safety profiles. The EVEREST II trial further identified functional MR as the strongest predictor of mortality (Hazard Ratio [HR] = 2.7 [95% Confidence Interval [CI] 1.4–5.0, *p* = 0.003]). Other factors associated with increased mortality risk included chronic obstructive pulmonary disease (COPD) (HR = 2.9 [95% CI 1.5–5.6, *p* = 0.001]), age (HR = 1.1 per 1-year increase [95% CI 1.0–1.1, *p* < 0.001]), diabetes mellitus (HR = 2.3 [95% CI 1.0–5.1, *p* = 0.05]), and peripheral arterial disease (HR = 2.1 [95% CI 0.99–4.5, *p* = 0.05]). Based on prior clinical trials, the 2025 ESC/EACTS Guidelines for the Management of Valvular Heart Disease [19] have substantially upgraded the recommendations for TEER in the interventional treatment of MR. For symptomatic severe secondary mitral regurgitation (SMR), the 2025 ESC Guidelines have upgraded TEER to a Class I recommendation (the highest level of recommendation) [26]. In patients with severe primary degenerative mitral regurgitation (DMR) who are at high surgical risk and have suitable anatomical characteristics, TEER was categorized as a Class IIb indication in the 2021 ESC Guidelines [27], and this recommendation has been updated to Class IIa in the new guidelines. For details, please refer to Section 9.1.4 of the new guidelines. Elderly patients with high-risk chronic DMR may benefit from minimally invasive TEER. For patients with atrial functional mitral regurgitation (AFMR) who remain symptomatic despite guideline-directed medical therapy (GDMT), TEER has been included in the indications for the first time (Class IIb recommendation, emerging recommendation level), while surgical intervention for such patients is recommended as a Class IIa indication. Relevant details can be found in Section 9.2.4 of the new guidelines.

##### 2.1.1.1 Key Updates Compared With Previous Guidelines

The recommendation level of TEER for patients with surgically high-risk primary mitral regurgitation (PMR) has been upgraded from Class IIb to Class IIa.

The recommendation level of TEER for patients with symptomatic ventricular secondary mitral regurgitation (vSMR) has been upgraded from Class IIa (Level B) to Class I (Level A).

The subsequent COAPT study [28] focused on the comparison between MitraClip and drug therapy. A total of 614 patients with heart failure and moderate-to-severe secondary MR who still had symptoms after receiving maximum-tolerated GDMT, were randomly assigned to the MitraClip + GDMT group (n = 302) and the GDMT alone group (n = 312). At 5-year follow-up, the rate of heart failure rehospitalization in the MitraClip + GDMT group was significantly lower than that in the GDMT alone group, and the all-cause mortality in the MitraClip + GDMT group was significantly lower than that in the GDMT alone group within 5 years. The results suggest that the 5-year outcomes of the cardiovascular outcomes assessment of the MitraClip percutaneous therapy (COAPT) study are generally positive, and the device can bring benefits to patients [29]. Another study, the MITRA-FR trial, yielded different outcomes compared with the COAPT trial [16]. It is hypothesized that the discrepancy between the two studies stems from differences in their inclusion criteria: MITRA-FR enrolled patients with less severe SMR, more significant left ventricular dilatation and dysfunction, and more advanced heart failure [16]. Although the MITRA-FR trial did not show that TEER combined with GDMT was superior to GDMT alone in endpoint outcomes, the COAPT-PAS study [30] emerged to fill this gap. This study evaluated the safety and efficacy of the MitraClip device in patients with heart failure and secondary MR in real-world settings, covering the research results of two subgroups of patients who met the criteria of the COAPT and MITRA-FR trials. Compared with patients receiving GDMT alone, patients receiving combined MitraClip therapy showed a similar or lower incidence of clinical events, similar rates of significant and sustained reduction in MR, and similar incidence of adverse events in the overall COAPT-PAS cohort, COAPT-like subgroup, and MITRA-FR-like subgroup. The application of MitraClip in real-world settings has significantly improved the quality of life and further reduced the rate of heart failure rehospitalization [31].

##### 2.1.1.2 Key Points

As the first TEER device approved by the three major authoritative institutions, MitraClip has established the clinical status of the TEER technology based on evidence-based medicine, promoted the upgrading of TEER recommendation levels in clinical guidelines, and set a benchmark for the research and development of similar devices in the future.

The eligible population includes patients with symptomatic severe vSMR; patients with severe primary DMR who are at high surgical risk and have suitable anatomical structures; and patients with AFMR who remain symptomatic despite receiving GDMT.

#### 2.1.2 The New-Generation MitraClip G4 Achieves Favorable Efficacy in Real-World Cohort Studies

The MitraClip G4 system has an improved clip arm design to achieve independent grasping of valve leaflets, and two wider models (NTW/XTW) have been added to meet the needs of TEER in MR patients with different anatomical structures. A Post-Market Study Assessment of the Safety and Performance of the MitraClip G4 System (EXPAND G4) study on the fourth-generation device is a prospective, multicenter, single-arm, real-world study, which included 1164 cases of primary or secondary MR treated with the MitraClip G4 system. The 30-day results [32] of the EXPAND G4 study demonstrated that the implantation success rate and acute procedural success rate were 98.0% and 96.2%, respectively. The device time and surgical time were significantly reduced compared with those in the EXPAND study. At 1 year [33], there was a durable reduction in MR to mild or less in 92.6% and to none or trace in 44.2% (*p* < 0.0001 vs. baseline). Few subjects had major adverse events through 1 year (<2% for myocardial infarction, surgical reintervention, or single-leaflet device attachment). The 1-year Kaplan-Meier estimates for all-cause mortality and heart failure hospitalization (HFH) were 12.3% and 16.9%. There were significant improvements in functional capacity, NYHA functional class I or II in 82%; *p* < 0.0001 vs. baseline, and quality of life (18.5-point KCCQ overall summary score improvement; *p* < 0.0001) were observed. The study showed that MV repair using the fourth-generation TEER device is a safe and effective treatment strategy, which not only reduces the degree of MR in patients with NYHA class IV acute heart failure but also improves clinical outcomes . After repair with the fourth-generation device, the subjects were divided into the HFH group and the non-HFH group. All groups maintained MR ≤1+, significant improvement in KCCQ score and NYHA classification at 1 year, and the 1-year all-cause mortality in the HFH group was significantly lower than that in the non-HFH group. Compared with the 1 year before treatment, the incidence of HFH in the 1 year after MitraClip treatment significantly decreased. The study showed that treatment with the fourth-generation device can significantly reduce HFH and improve patients’ symptoms and quality of life [34].

##### Key Points

The optimized clip arm design and newly added models of the MitraClip G4 System have improved its adaptability to different anatomical structures, shortened the surgical time, enhanced the operational efficiency, and further expanded the application of the TEER technology.

It is suitable for patients with primary or secondary MR with various anatomical structures, including patients with NYHA Class IV acute heart failure, and patients with wide valve leaflets who are ineligible for the conventional MitraClip models.

The efficacy in patients with extremely complex anatomical structures still needs to be further verified. The durability of long-term (more than 1 year) efficacy and device longevity remains to be confirmed by longer-term follow-up, and the prognosis of some elderly patients with multiple comorbidities is still unsatisfactory.

#### 2.1.3 Indications for TEER Technology Continue to Expand, Achieving Breakthroughs in Unsuitable Anatomical Structures and Heart Failure

**Fig. 2.**
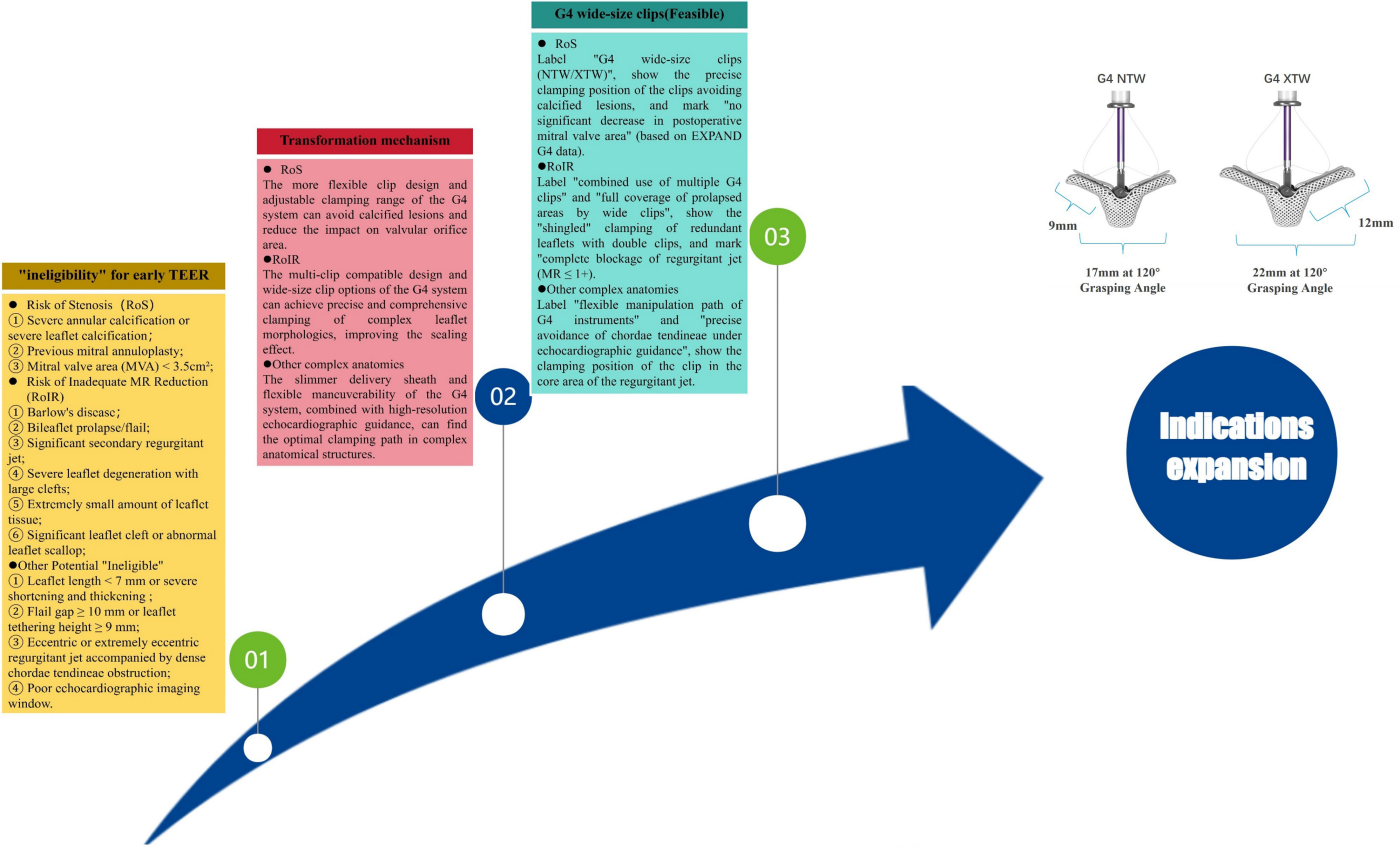
**Anatomical transition from "ineligible" to "eligible"**.

In the early stage, due to the lack of experience, TEER technology was proposed for "unsuitable" anatomical and clinical criteria. However, based on the echocardiographic and clinical results of the EXPAND G4 real-world post-marketing study, the scope of TEER suitability was explored (Fig. 2). According to the definition of the Heart Valve Collaboration TEER unsuitability criteria, three groups were defined: (1) risk of stenosis (RoS); (2) risk of insufficient MR reduction(RoIR); (3) subjects with baseline MR of moderate or lower (MMR). In addition, those without these characteristics were defined as the TEER suitable (TS) group . All groups had a high 30-day MR reduction rate (≤1+), safely achieved 30-day NYHA functional improvement (RoS 94% vs. 29%, RoIR 88% vs. 30%, MMR 79% vs. 26%, TS 83% vs. 33%) and quality of life improvement (RoS +27 ±26, RoIR +16 ±26, MMR +19 ±26, TS +19 ±24), with low rates of major adverse events and all-cause mortality. The study showed that with the continuous progress of techniques and devices, patients previously considered unsuitable for TEER treatment can be safely and effectively treated with the fourth-generation device [35]. The RESHAPE-HF2 study is a prospective, randomized, parallel-controlled, multicenter clinical trial aimed at evaluating the safety and efficacy of the MitraClip device combined with optimal standard therapy (Device Group) versus optimal standard therapy alone (Control Group) in the treatment of MR [36]. The patients in this trial represent the third unique population in the MitraClip trials, which consists of patients with moderate to severe functional FMR, while only moderate to severe patients were included in the COAPT and MITRA-FR trials . The results of the study showed that the proportion of patients with MR ≤2+ in the MitraClip device group at 12 months was significantly higher than that in the control group. The composite risk of HFH and cardiovascular death was significantly reduced, the rate of heart failure rehospitalization was significantly decreased, and the quality of life was significantly improved. The successful application of this trial provides a new treatment option for patients with heart failure and FMR, especially those who cannot accept or are unwilling to undergo open-heart surgery. The RESHAPE-HF2 study has promoted technological progress in the field of heart failure treatment and provided new ideas for the design of future clinical trials and treatment strategies [37].

##### Key Points

It breaks through the limitations of ineligible patient populations associated with early TEER technologies, expands the indications to patients with moderate-to-severe FMR, and provides minimally invasive treatment opportunities for more patients with MR.

It is suitable for the following MR patients: those previously deemed anatomically ineligible for TEER treatment; patients with moderate-to-severe functional FMR who remain symptomatic despite GDMT; and heart failure patients complicated with FMR who are unable or unwilling to undergo surgery.

The long-term benefits for patients with baseline moderate or less severe MR remain unclear; there is still room for improvement in the surgical success rate among patients with complex anatomical structures; and long-term efficacy data for subgroups of FMR patients with different etiologies are lacking.

#### 2.1.4 TEER Devices Continue to Upgrade, Covering Populations With Various Etiologies of MR and Complex Anatomical Structures

PASCAL is another trans-femoral vein-transseptal MV clipping system based on the "Alfieri Stitch" technique. Its clipping arms are wider and longer than those of the traditional MitraClip, and can be independently clamped. The isolation ball in the middle is beneficial to reduce valve tension. PASCAL obtained CE certification in 2020 and has performed well in the European market. PASCAL Precision received FDA approval in 2022 for the treatment of DMR patients. The CLASP IID randomized controlled trial, a clinical study of PASCAL Precision, is a prospective, multicenter randomized controlled trial that included 300 patients with 3+ or 4+ DMR and surgical contraindications from 57 centers in multiple countries, randomly divided into the PASCAL group and the MitraClip group. At 1-year follow-up, there were no statistically significant differences in all-cause mortality, heart failure rehospitalization, and major adverse event rates between the PASCAL group and the MitraClip group. 95.8% of patients in the PASCAL group had MR ≤2+ at 1 year after surgery, which was not inferior to the MitraClip group. Both groups had significant improvements in cardiac function classification and quality of life. The results of the study confirmed that the PASCAL system is an effective treatment device for patients with severe DMR who have increased surgical risk and symptoms [38]. The PASCAL IID trial is a single-arm trial belonging to CLASP IID, which evaluated the safety, echocardiographic, and clinical outcomes of PASCAL and MitraClip in patients with severe DMR and complex MV anatomy who have surgical contraindications. The implantation success rate was 92.9%, and the 30-day composite major adverse event rate was 11.2%. At 6-month follow-up, the proportion of patients with MR ≤2+ was 97.7% in the PASCAL group and 98.1% in the MitraClip group, achieving statistical non-inferiority. The proportion of patients with MR ≤1+ was 83.7% in the PASCAL group and 71.2% in the MitraClip group, with the PASCAL group showing a trend of being superior to the MitraClip group. There was no statistically significant difference in major adverse events between the two groups (3.4% in the PASCAL group vs. 4.8% in the MitraClip group). In addition, left ventricular remodeling and improved cardiac function were observed in both groups, with no statistically significant differences between the groups. The study confirmed that the PASCAL system is a treatment option suitable for DMR patients with complex MV anatomy and high surgical risk [39].

The MiCLASP Study is a prospective, multicenter clinical study aimed at evaluating the safety and efficacy of the Edwards PASCAL device in the treatment of MR patients with significant clinical symptoms. Following its marketing in Europe, 59% of the patients had functional MR, and 30% had degenerative MR. The results of the study showed that the 30-day incidence of composite major adverse events was 6.8%, including cardiovascular mortality (1.1%), stroke (0.9%), myocardial infarction (0.2%), MV reintervention rate (1.3%), major cardiac structural complications (0.4%), device embolization (0.2%), renal complications requiring dialysis (1.1%), severe bleeding (4%), and major puncture site and vascular complications (1.1%). Compared with baseline, MR was significantly reduced at discharge and remained stable at 1 year, with 98% of patients having MR ≤2+ and 82.6% having MR ≤1+. The 1-year Kaplan-Meier survival rate was 87.3%, and the rate of no HFH was 84.3%. In addition, patients’ function and quality of life were significantly improved within 1 year. The study showed that the PASCAL system is safe and effective in the treatment of FMR and DMR, which provides strong evidence for TEER as a feasible option for the treatment of MR [40].

##### Key Points

The unique clip arm design and isolation balloon structure of PASCAL improve the adaptability to complex anatomical structures, provide a novel therapeutic option for patients with FMR and DMR, and form a complementary alternative to MitraClip.

It is indicated for the following patients: symptomatic patients with severe DMR who are at excessively high surgical risk; patients with severe DMR accompanied by complex MV anatomy, and patients with FMR.

Comparative data regarding the durability of long-term efficacy between PASCAL and MitraClip are limited; the therapeutic effect in patients with extremely severe complex anatomical structures needs further verification; and there is a lack of dedicated studies on patients with multiple comorbidities.

#### 2.1.5 Domestic Devices Show an Increasing Trend, With a Number of Innovative Devices With Different Pathways Emerging

ValveClamp is the first domestic transapical TEER device to be marketed. The marketing of this device is based on the results of the multicenter CLAMP-2 study, which showed an acute surgical success rate of 97%, 88% of patients only needed to implant one clip device to achieve the treatment goal, and the effective endpoint at 1 year after surgery was 87.3% [41]. DragonFly is China’s first trans-femoral vein edge-to-edge repair device, which was officially approved for marketing by NMPA in November 2023. The DRAGONFLY-DMR trial, aimed at evaluating the safety and efficacy of the DragonFly system, is a prospective, single-arm, multicenter clinical study that included 120 patients with clinical symptoms, MR ≥3+, and high surgical risk DMR from 27 centers in China. The clinical success rate at 1 year was 87.5%, and the rates of major adverse events, all-cause mortality, MV reintervention, and HFH were 9.0%, 5.0%, 0.8%, and 3.4%, respectively. The rates of MR ≤2+ at 1 month and 1 year were 90.4% and 92.0%, respectively, and patients’ cardiac function and quality of life were significantly improved. The study showed that the DragonFly system helps to provide more evidence supporting the safety and efficacy of TEER therapy for the treatment of chronic symptomatic 3+ to 4+ DMR patients at high surgical risk [42].

##### Key Points

ValveClamp adopts a transapical approach, while DragonFly uses a transfemoral venous approach. These two devices expand the options for TEER devices in China and are more compatible with the anatomical characteristics and clinical needs of Chinese patients.

ValveClamp is indicated for high risk surgical patients with FMR or DMR, especially those with restricted transfemoral venous access; DragonFly is indicated for symptomatic high-surgical-risk patients with DMR and MR ≥3+.

Long-term efficacy and durability data of the two devices still need to be obtained; clinical experience in treating MR patients with complex anatomical structures and special etiologies is insufficient; head-to-head comparative studies with imported devices are relatively scarce.

#### 2.1.6 The Efficacy of the TEER-Specific Risk Scoring System Is Superior to Traditional Risk Scores

With the rapid advancement of the application of TEER, the risk assessment of TEER surgery has attracted increasing attention. The MitraScore [43] is a scoring system specifically used for risk stratification of TEER surgery, based on data from 1119 TEER surgeries in the multinational international registry study MIVNUT. The MitraScore includes 8 independent predictors of patient death: age ≥75 years, anemia, glomerular filtration rate <60 mL/min/1.73 m^2^, left ventricular ejection fraction (LVEF) <40%, peripheral artery disease, chronic obstructive pulmonary disease, high-dose diuretics (≥80 mg furosemide/day or use of ≥2 diuretics other than anti-aldosterone drugs), and no use of renin-angiotensin system inhibitor therapy. Each factor is assigned 1 point, and for each additional point, the relative risk of all-cause death in patients increases by 55% (HR 1.55, 95% CI 1.44–1.67; *p* < 0.001). The overall mortality rate increases by 20 times from the lowest score to the highest score. The discrimination and calibration effects of this score in predicting mortality are superior to the current EuroSCORE Ⅱor STS scores. Thus, the MitraScore is a simple prediction algorithm that can be used to predict the mortality of patients undergoing TEER treatment.

## 3. Other Transcatheter Mitral Valve Repair Technologies

Compared to TEER, MV annuloplasty has increased technical barriers and operational difficulty, and a long learning curve, so its development is relatively slow. According to different mechanisms, annuloplasty can be divided into direct annuloplasty and indirect annuloplasty. Currently, marketed products include the direct annuloplasty device Cardioband and the indirect annuloplasty device Carillon. Cardioband is a direct annuloplasty device delivered via transseptal puncture, which obtained European CE certification in 2015. A one-year follow-up of 60 patients with moderate to severe FMR from 11 institutions in Europe treated with the Cardioband System showed that the 1-year overall survival rate, survival rate without heart failure re-admission, and survival rate without reintervention were 87%, 66%, and 78%, respectively. 95% of patients who underwent transthoracic echocardiography at 1 year had moderate or less MR, and NYHA classification, quality of life, and exercise capacity were significantly improved [44]. Carillon is an indirect annuloplasty device placed in the coronary sinus, which reduces FMR by narrowing the annulus. The REDUCE-FMR study showed that Carillon implantation reduced the regurgitant volume and left ventricular volume in patients with severe FMR [45]. DragonRing is China’s first trans-femoral vein interventional annuloplasty system that simulates atrioventricular annuloplasty technology and is independently developed. It can be used for patients with functional mitral and tricuspid regurgitation caused by annulus dilatation, leading to the loss of leaflet coaptation height. The world’s first human implantation was completed by Professor Chen Mao’s team [46].

Due to technical difficulties, transcatheter chordae repair devices are currently in animal experiments or early clinical stages, including NeoChord NeXuS, CardioMech, and Pipeline. Transcatheter chordae repair devices consist of an implant and a delivery system. The implant includes an artificial chordae suture device, a papillary muscle anchor piece, and leaflet anchor pieces connected at both ends. Rogers *et al.* [47] first reported a human trial in 2020, but the papillary muscle anchor piece fell off before discharge, and the patient finally underwent MV replacement. Latib *et al.* [48] completed the first FIFH clinical trial and reported results at 6-month follow-up, which successfully reduced MR, ensuring that the papillary muscle was firmly anchored without displacement. Transcatheter chordae repair devices may be a feasible option for the treatment of isolated single-leaflet prolapse in the future, with a good risk-benefit ratio.

### Key Points

Annuloplasty targets annular dilatation, the key pathological mechanism, offering a precise treatment strategy for patients with FMR. Chordal repair devices simulate the anatomical repair concept, which is expected to achieve curative treatment for leaflet prolapse and enrich the technical system of TMVr.

Annuloplasty is indicated for patients with FMR caused by annular dilatation, especially those with suboptimal outcomes after TEER. Chordal repair devices are indicated for patients with DMR induced by isolated single-leaflet prolapse.

Annuloplasty involves complex procedures and a long learning curve, making it difficult to promote in primary hospitals. Chordal repair devices are still in the early stage of research and development, and their long-term efficacy, device durability, and safety require further verification in more clinical trials.

## 4. Transcatheter Mitral Valve Replacement

**Fig. 3.**
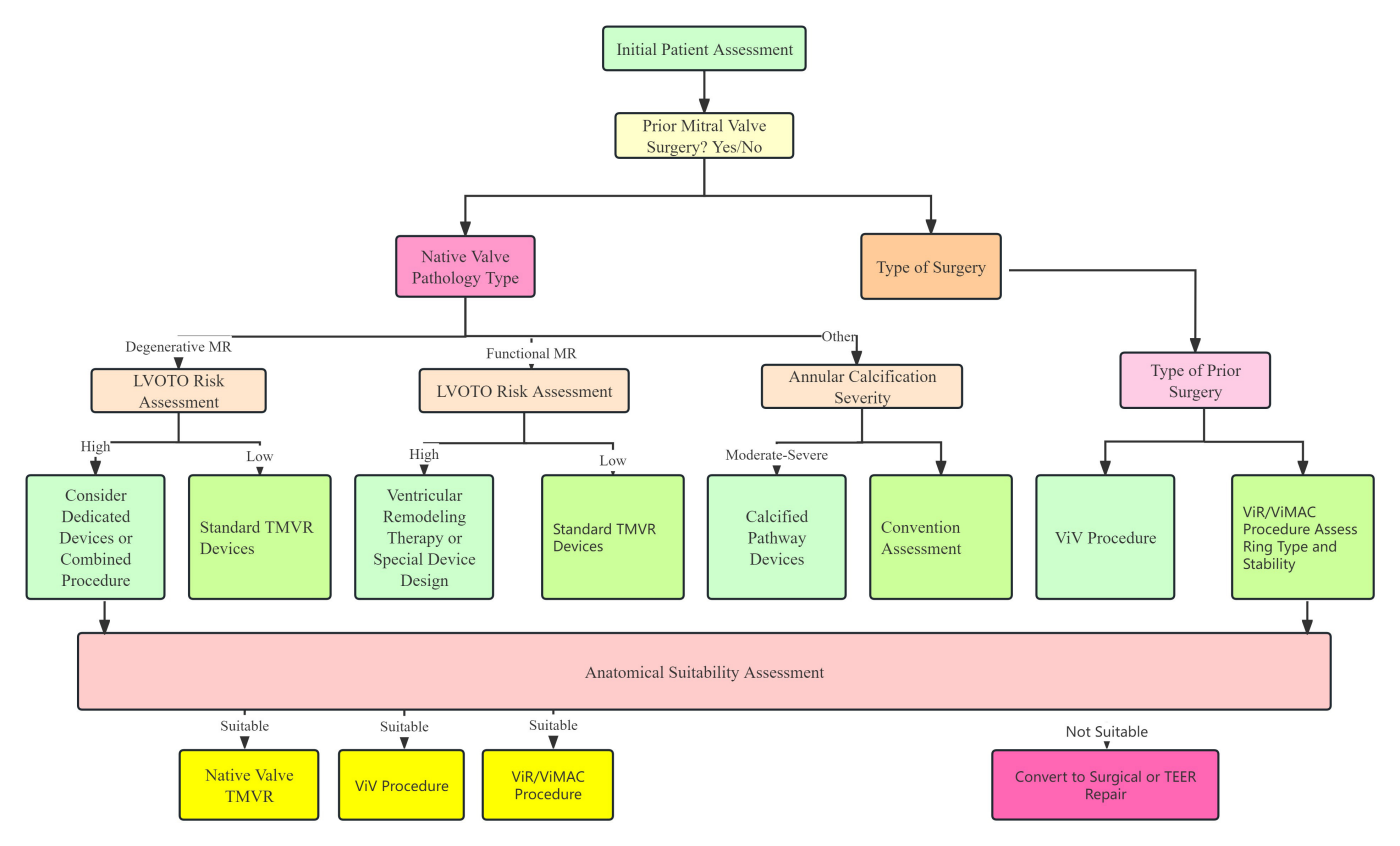
**Flowchart of TMVR procedure selection**.

Compared with TEER, TMVR has the advantage of being able to completely cure MR. The flowchart for the selection of various TMVR procedures is shown in Fig. 3. According to the type of native MV, TMVR can be divided into four types: (1) valve-in-valve (ViV) after bioprosthesis failure; (2) valve-in-ring (ViR); (3) valve-in-calcified annulus (ViMAC); (4) valve-in-native valve.

### 4.1 TMVR Has a Wide Range of Applications and Shows Excellent Therapeutic Effects in Different Clinical Settings

#### 4.1.1 ViV, ViR, and ViMAC TMVR Are Reasonable Choices in Patients at High Surgical Risk

The MITRAL trial is the first prospective study aimed at evaluating the early feasibility of transseptal ViV, ViR, and ViMAC using balloon-expandable valves SAPIEN XT/3. A total of 91 patients with severe mitral stenosis or MR, NYHA class II or higher, or assessed as high surgical risk by the heart team were included and assigned to three treatment strategies: ViV (N = 30), ViR (N = 30), and ViMAC (N = 31). The 5-year follow-up results showed that the all-cause mortality rates in the ViV, ViR, and ViMAC groups were 21.4%, 65.5%, and 67.9%, respectively, and the proportions of NYHA class I–II were 94.7%, 50%, and 55.6%, respectively. The Kansas City Cardiomyopathy Questionnaire scores in the three groups continued to improve compared with baseline, and the function of mitral bioprostheses in all groups remained stable. The study showed that ViV, ViR, and ViMAC are reasonable choices for MV patients at high surgical risk [49].

#### 4.1.2 Compared With TEER, Patients With Severe FMR Undergoing TMVR Have Better MR Reduction and Symptom Improvement

The CHOICE-MI study conducted a propensity score matching analysis between 262 FMR patients who underwent TMVR and 1065 FMR patients who underwent TEER in the EuroSMR registry study. The results showed that there was no statistically significant difference in all-cause mortality between TMVR and TEER at 30 days and 1 year. TMVR had better MR reduction (the proportions of patients with residual MR ≤1 at discharge in TMVR and TEER groups were 95.8% and 68.8%, respectively, *p* < 0.001) and symptom improvement (the proportions of patients with NYHA ≤II at 1 year were 77.8% and 64.3%, respectively, *p* < 0.001). The study showed that TMVR is associated with better MR reduction and symptom improvement [50].

#### 4.1.3 Compared With GDMT Alone, FMR Patients Undergoing TMVR Have Better MR Reduction and Symptom Improvement

A propensity score matching analysis aimed at comparing the effects of TMVR and GDMT in the treatment of MR included 97 FMR patients who underwent TMVR in the CHOICE-MI registry study and 97 patients who received GDMT in the COAPT trial. The results showed that the 2-year heart failure rehospitalization rate in the TMVR group was significantly lower than that in the GDMT group (32.8% vs. 54.4%, HR 0.59, 95% CI 0.35~0.99, *p* = 0.04). All patients in the TMVR group had residual MR ≤1+ at 1 year and 2 years, while those in the GDMT alone group were 6.9% and 7.7%, respectively. The proportions of patients in NYHA class I or II in the TMVR group were higher than those in the GDMT group at 1 year (78.2% vs. 59.7%, *p* = 0.03) and at 2 years (77.8% vs. 53.2%, *p* = 0.09). The 2-year mortality rates in the two groups were similar (36.8% vs. 40.8%; HR 1.01, 95% CI 0.62~1.64, *p* = 0.98). The study showed that compared with GDMT, TMVR can significantly reduce the degree of MR, improve symptoms, and reduce the rate of heart failure rehospitalization [51].

#### 4.1.4 TMVR Shows Lower Initial Risk After Mitral Bioprosthesis Failure

Currently, evidence on the prognosis of redo surgical MV replacement (Redo SMVR) or TMVR after mitral bioprosthesis failure is limited, and a propensity score matching analysis fills this gap. A total of 4293 patients were included in the study (redo SMVR: 64%; TMVR: 36%). Landmark analysis showed that TMVR had a lower risk of MACE in the first 6 months (adjusted HR: 0.75; 95% CI: 0.63~0.88; *p* < 0.001), but a higher risk after 6 months (adjusted HR: 1.28; 95% CI: 1.04~1.58; *p* = 0.02). The study showed that the 3-year prognosis of Redo SMVR and TMVR is similar, with TMVR showing a lower initial risk but a higher risk of MACE after 6 months [52]. Patients undergoing ViV-TMVR are mostly at high surgical risk, with an average Society of Thoracic Surgeons (STS) score >8 points. Short-term outcomes show that the 30-day mortality rate ranges from 3.2% to 7.5%, the incidence of stroke is <3%, and the incidence of postoperative left ventricular outflow tract (LVOT) obstruction is relatively low. Long-term outcomes indicate a 1-year mortality rate of approximately 15% and a 4-year mortality rate of 37.5% [53,54,55,56,57,58,59]. High STS score, advanced age, poor left ventricular function, and moderate to severe tricuspid regurgitation before surgery are the main factors associated with increased long-term all-cause mortality.

The mitral annulus has a saddle-shaped structure with a three-dimensional and dynamically changing morphology. In patients with MR, the annulus often exhibits mild or no calcification. These factors usually hinder the anchoring of interventional devices, leading to poor adherence and thereby increasing the risks of device embolization and paravalvular leak. In addition, the complex anatomical morphology of the subvalvular apparatus of the MV further increases the difficulty of interventional procedures. LVOT is the most feared complication of ViV-TMVR and is an independent predictor of early adverse outcomes in patients [60]. Compared with surgeries involving transcatheter MV-in-ring implantation or mitral annular calcification (MAC), the incidence of LVOT obstruction in ViV-TMVR is lower (≤5%). The reasons include: the anterior leaflet has been resected during the original MV replacement; most surgical bioprosthetic valves are visible under fluoroscopy, enabling precise positioning of the transcatheter heart valve; and surgical bioprosthetic valves can serve as an anchoring system for the new valve, avoiding valve displacement or embolization.

##### Key Points

Compared with the palliative treatment effect of TEER, TMVR can completely correct MR, offering a more effective therapeutic option for patients with severe FMR, especially those with suboptimal MR relief after TEER.

It is indicated for patients with severe FMR, particularly those with anticipated poor TEER outcomes or unsuitable anatomical conditions for TEER, and FMR patients who still present with obvious symptoms despite GDMT.

The surgical procedure is more technically demanding than TEER with a longer learning curve; the risks of postoperative complications, such as paravalvular leak and LVOTO, are slightly higher than those of TEER; the long-term efficacy, durability, and device longevity need further verification.

### 4.2 A Variety of TMVR Devices Have Entered Clinical Trials and Achieved Favorable Preliminary Follow-up Results

TMVR devices are diverse in type and at different stages of research and development, as illustrated in Table 2. The Tendyne Mitral Valve Replacement System was the first commercially available TMVRdevice approved in Europe in 2020. The Tendyne System has the most extensive clinical data among TMVR devices; existing research [61] has demonstrated that this system is safe and feasible, and enables the complete elimination ofMR. In addition, several relevant clinical studies are currently underway, including the SUMMIT Trial (NCT03433274), the Mitral Annular Calcification Study (NCT03539458), and the Expanded Access Study (NCT02321514). In addition, the 1-year follow-up data from the investigator-initiated TENDER registry in real-world settings showed that the 30-day cardiovascular mortality and all-cause mortality of Tendyne TMVR were 7% and 9%, respectively, and the 1-year cardiovascular mortality and all-cause mortality were 17% and 29%, respectively. The rate of reintervention or surgery after discharge was 4%, while the rate of heart failure rehospitalization decreased from 68% to 25%. 98% of patients achieved sustained reduction of MR to ≤1+, and 83% of patients achieved NYHA functional class I or II at 1 year. This large-scale, real-world observational registry study reported that Tendyne TMVR has a high technical success rate, lasting and complete elimination of MR, and significant clinical benefits, and that the results of on-label use are comparable to those in "real-world" use, providing a safe and effective treatment option for patients who previously had no alternative therapy [62].

**Table 2.** **TMVR devices in clinical trial phase**.

Device	Medical device company	Stent	Valve leaflet	Delivery system	Access route	Anchoring mechanism	Illustration
AltaValve	4C Medical Technologies (USA)	Self-expanding nitinol alloy	Bovine pericardial trileaflet valve	32 F	Transapical, Transfemoral	Spherical stent, supra-annular deployment	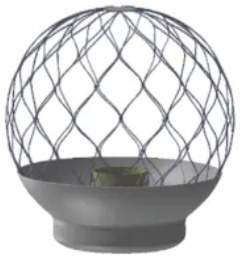
Cardiovalve	Cardiovalve (Israel)	Self-expanding type	Bovine pericardial trileaflet valve	28 F	Transfemoral	Intra-annular anchoring	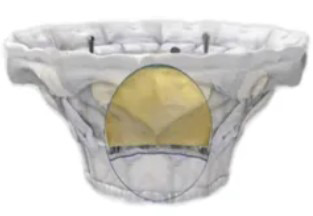
Cephea	Gore (USA)	Self-expanding nitinol alloy, double-layer disc-shaped	Bovine pericardial trileaflet valve	32 F	Transfemoral	Axial tension	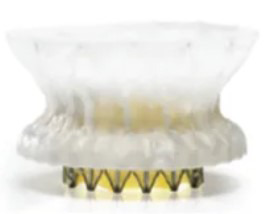
Evolve	Edwards Lifesciences (USA)	Self-expanding nitinol alloy	Bovine pericardial trileaflet valve	28 F	Transfemoral	Multi-layer anchoring (annulus, leaflet, sinus)	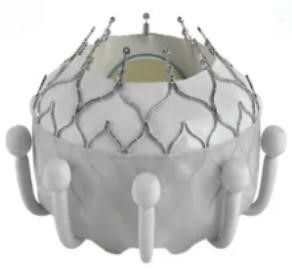
Highlife	HighLife SAS (USA)	Self-expanding nitinol alloy; sub-annular implant	Bovine pericardial trileaflet valve	39 F	Transapical, Transfemoral	Sub-annular fixation	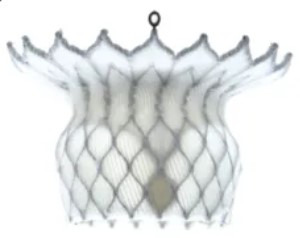
Intrepid	Medtronic (USA)	Self-expanding nitinol alloy, double-layer stent	Bovine pericardial trileaflet valve	35 F	Transapical, Transfemoral	Staple-gripped leaflet	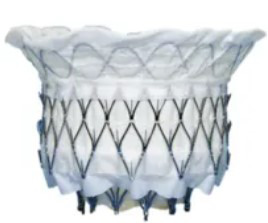
Sapien M3	Edwards Lifesciences (USA)	Spherical cobalt-chromium alloy	Bovine pericardial trileaflet valve	20 F	Transfemoral	Self-expanding base	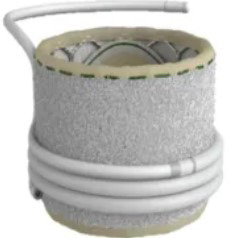
Tendyne	Gore (USA)	Self-expanding double-layer nitinol alloy	Porcine pericardial trileaflet valve	34~36 F	Transapical	Apical suture anchored to epicardium	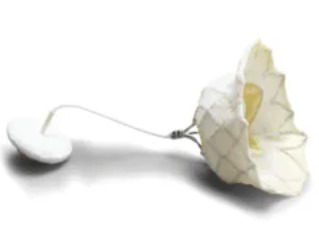
Tiara	Neovasc (Canada)	Self-expanding nitinol alloy	Bovine pericardial trileaflet valve	32, 36 F	Transapical	Ventricular anchoring device	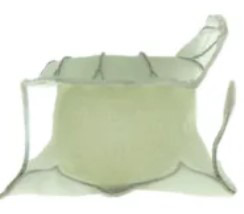

HighLife is a transseptal MV replacement system with a unique "valve-in-ring" design, featuring self-adaptation and self-coaxiality. The results of an expanded study on the HighLife system in patients with severe MR who are unsuitable for surgery or transcatheter repair showed that the technical success rate of HighLife TSMVR system implantation in 30 patients was 90%, the device success rate within 30 days was 83%, and the mortality rate at 1 year was 17%. All implanted patients had no significant or mild MR, the mean postoperative pressure difference was 5.1 mmHg, and there was no left ventricular outflow tract obstruction (LVOTO). The study results showed that this technology has a high success rate, good valve function, no LVOTO, and no need for further MV intervention [63].

Intrepid is one of the most widely implanted valves clinically. The results of a two-year clinical and echocardiographic follow-up of the transapical Intrepid System implantation showed that the all-cause mortality rates at 30 days, 1 year, and 2 years were 13.1%, 27.3%, and 36.2%, respectively, and the rates of heart failure rehospitalization at 30 days and 2 years were 9.6% and 36.2%, respectively. More than 50% of patients survived at 2 years with improved NYHA functional classification, and all patients who underwent echocardiography had MR ≤ mild. This study is the largest reported experience of transapical TMVR and the longest follow-up analysis of high-risk patients with MR ≥ moderate to severe. Although early mortality and heart failure rehospitalization rates were high, clinical outcomes improved and MR severity decreased significantly within 2 years [64].

The research and development of TMVR devices in China lagged behind that in other countries. To date, more than 10 types of TMVR valves have been developed, among which 6 have entered the clinical research phase (Table 3). Relevant early feasibility studies and confirmatory trials for further assessment of safety and efficacy are currently underway. Among these devices, the MitraFix, Mi-thos, TruDelta, and AltaValve systems have been reported in several case series, demonstrating favorable clinical outcomes. The one-year follow-up results of the MitraFix study showed a 100% procedural success rate. MR was completely eliminated in 90% of patients after surgery, while the remaining patients had their MR reduced to grade 1+. Moreover, 75% of the surviving patients achieved improvement in New York Heart Association (NYHA) functional class to grade I or II. No complications such as LVOTO or mitral stenosis were observed during the follow-up period, and the 6-minute walk distance was significantly increased. These findings indicate that the MitraFix system exhibits increased advantages in terms of procedural success rate and patient prognosis, providing a novel, safe, and effective therapeutic option for high-risk patients with severe MR [65].

**Table 3.** **China’ s investigational TMVR devices**.

Device	Medical enterprise	Stent	Valve leaflet	Delivery system	Access route	Anchoring mechanism	Schematic
MitraFix	Yixin Medical	Nitinol	Bovine Pericardial Trileaflet Valve	30 F	Transapical, Transfemoral-Transseptal	Anchoring Claws and D-shaped Umbrella Structure on Atrial Side	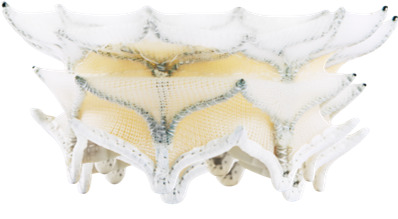
Mi-thos	Newmed Medical	Nitinol	Bovine Pericardial Trileaflet Valve	36 F	Transapical	Barbs + Axial Tension	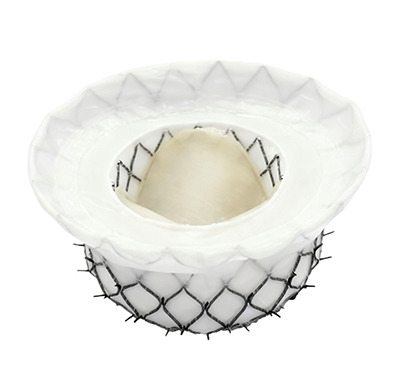
TruDelta	Zhenyi Medical	Nitinol	Biological Valve Leaflet	33 F	Transapical	Distal Suture	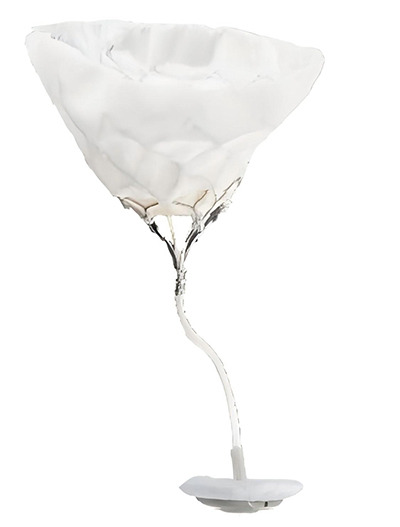
Cardiovalve	Qiming Medical	Self-Expandable	Bovine Pericardial Trileaflet Valve	28 F	Transfemoral-Transseptal	Intra-Annular Anchoring	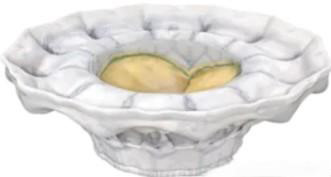
AltaValve	Xintong Medical & 4C Medical Technologies (USA)	Self-Expandable Nitinol	Dry Valve	29 F	Transapical, Transfemoral-Transseptal	Atrial Anchoring	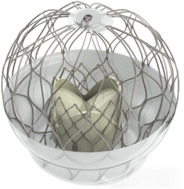
Highlife	Pujia Medical	Self-Expandable Nitinol	Bovine Pericardial Trileaflet Valve	39 F	Transapical, Transfemoral-Transseptal	Intra-Valvular Annular Anchoring	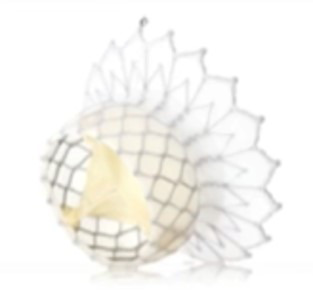

#### Key Points

Various TMVR devices address the anatomical challenges of native MV replacement through a unique design, which improves the surgical success rate and safety and promotes the popularization of TMVR technology.

It is indicated for high-surgical-risk patients with severe MR, including those with DMR and FMR; patients with complex anatomical MR who are ineligible for surgical treatment or transcatheter repair; Chinese devices are applicable to Chinese MR patients who meet the enrollment criteria of clinical studies.

Most devices are still in the clinical trial phase with limited data on long-term efficacy and durability; the risk of postoperative complications associated with some devices still needs to be reduced; clinical data of Chinese devices is insufficient, and there are relatively few comparative studies with other international devices.

## 5. Imaging Evaluation and Intraoperative Operational Strategies

Imaging evaluation is the core support for transcatheter intervention in MR, running through the entire process of preoperative plan formulation, intraoperative precise operation, and postoperative efficacy assessment. Transesophageal echocardiography (TEE) and computed tomography (CT) serve as key imaging tools, and their accurate measurement results directly drive the selection of treatment plans. The key intraoperative verification points are the core links to ensure surgical success and reduce the risk of complications.

Preoperatively, multi-dimensional and multi-parameter measurements using TEE and CT are required to comprehensively assess the anatomical characteristics of the heart, providing support for treatment plans and device selection. TEE excels in evaluating valve function, leaflet motion, and hemodynamics, while CT has advantages in three-dimensional anatomical reconstruction, precise size measurement, and assessment of calcification. The core measurement indicators are detailed in Table 4 [66].

**Table 4.** **Core preoperative measurement indicators**.

Evaluation dimension	Specific indicators	Measurement tools	Normal range/reference standards	Clinical significance and treatment decision guidance
Annulus-related	3D diameter (anteroposterior diameter/intercommissural diameter/mean diameter)	CT as the main tool, TEE as auxiliary	Mean diameter: 20–35 mm	>35 mm indicates significant annular dilatation, favoring annuloplasty or large-sized devices; in TMVR, precise matching with prosthesis size is required to avoid LVOTO or paravalvular leak
	Circumference and area	CT 3D reconstruction	-	Evaluate the degree of annular dilatation to guide the anchoring site and tightening amplitude of annuloplasty devices
	Calcification degree (range/thickness/distribution)	CT (gold standard)	Thickness: mild <2 mm, moderate 2–4 mm, severe >4 mm	Severe diffuse calcification → prioritize TMVR; localized posterior annular calcification → TEER combined with annuloplasty is feasible
Leaflet-related	Leaflet length (anterior leaflet/posterior leaflet)	TEE	Anterior leaflet: 20–25 mm, posterior leaflet: 15–20 mm	<18 mm (anterior)/<12 mm (posterior) → select wide-jaw devices (e.g., MitraClip G4 XTW); >28 mm (anterior) → prioritize TEER or artificial chordae repair
	Leaflet coaptation gap	TEE (diastolic phase)	≥5 mm	<3 mm (functional MR) → ideal indication for TEER; <1 mm → consider TMVR
	Thickness and prolapse range	TEE (3D)	Thickness <3 mm; prolapse <10 mm (localized), >10 mm (extensive)	Thickness >4 mm → indicates fibrosis; localized prolapse → TEER/artificial chordae repair; extensive prolapse → consider TMVR
Calcification morphology	Leaflet calcification (location/range/thickness)	TEE + CT	-	Severe free-edge calcification → TEER should be performed cautiously; body calcification involving the coaptation zone → favor TMVR
	Left ventricular outflow tract calcification	CT	-	Severe calcification → formulate calcification management plan (e.g., balloon pre-dilation) before TMVR
TMVR-specific anatomy	Aortic-mitral valve angle (AMA)	CT 3D reconstruction	120°–150°	<120° (narrow angle) → select low-profile valves to prevent LVOTO; >150° (wide angle) → select devices with strong anchoring design
Auxiliary evaluation	Left ventricular outflow tract diameter (LVOTD)	CT	20–25 mm	<20 mm → high risk of postoperative LVOTO in TMVR, requiring strict control of valve size
	Mitral annulus-left ventricular apex distance (A2A)	CT	45–60 mm	<45 mm → high risk of LVOTO; >60 mm → increased risk of valve displacement
	Coronary artery anatomy	CT coronary angiography	-	Left circumflex artery ostium distance from annulus <5 mm → avoid compression risk during TMVR

CT, computed tomography; TEE, Transesophageal echocardiography.

Intraoperatively, real-time imaging monitoring and operational verification should be combined to ensure accurate device positioning, good valve function, and reduce the risk of immediate complications. Key verification points are confirmed in stages according to the operational process, as detailed in Table 5 [67].

**Table 5.** **Verification of puncture path and device placement**.

Verification link	Core points	Monitoring tools	Qualified Standards
Transfemoral venous-interatrial septal puncture	Puncture site location, size of atrial septal defect	TEE + angiography	Puncture site located in the middle-upper part of the interatrial septum, no leaflet/atrial wall injury; defect size adapted to the delivery sheath, no significant bleeding
Transapical approach	Apical puncture site, stability of fixation device	Fluoroscopy + TEE	Puncture site located at the center of the left ventricular apex, firmly fixed without displacement
Device placement verification	Angle between device axis and mitral annulus plane	TEE	Device axis is perpendicular to the mitral annulus plane without inclination/displacement

TTE is routinely used for follow-up after TEER. When TTE imaging is limited, device-related complications are suspected, or MR worsening requires precise assessment of pathological mechanisms, further TEE can be performed and compared with intraoperative data. Common evaluation indicators are detailed in Table 6 [66]. Clip stability is also an important part of postoperative TEER evaluation, and its assessment points are detailed in Table 7 [68].

**Table 6.** **TEER Postoperative MR Grading Indicators**.

Parameters	Mild (1+)	Moderate (2+)	Severe (3+/4+)
Qualitative evaluation			
Color Doppler regurgitant jet	1–2 thin, narrow jets	Between mild and severe	Wide central regurgitant jet; Multiple regurgitant jets; Atrial wall-adherent eccentric regurgitant jet
Flow convergence zone size	Absent or small (PISA radius ≤3 mm)	Moderate (PISA radius 3 mm–9.9 mm)	Large (PISA radius ≥10 mm)
Mitral inflow spectral morphology	A-wave dominant	No specific indicators	No specific indicators
Pulmonary venous flow spectrum	Normal S-wave	Blunted S-wave	Reversed S-wave
Continuous wave Doppler spectral morphology	Blurred parabolic contour	-	Dense triangular contour
Semi-quantitative evaluation			
VCW	Single regurgitant jet and VCW ≤0.3 cm	Single regurgitant jet and VCW 0.4 cm–0.6 cm	Any regurgitant jet with VCW ≥0.7 cm or ≥2 moderate or above regurgitant jets
Quantitative evaluation			
VCA (3D)	Single regurgitant jet and VCA ≤0.2 cm^2^	Single regurgitant jet and VCA 0.2 cm^2^–0.39 cm^2^	Any regurgitant jet with VCA ≥0.4 cm^2^ or ≥2 moderate or above regurgitant jets
PISA method EROA (cm^2^)	<0.2	0.2–0.39	≥0.4
RVol (mL)	<30	30–59	≥60
RF (%)	<30	30%–49%	≥50

VCW, vena contracta width; VCA, vena contracta area; PISA, proximal isovelocity surface area; RVol, regurgitant volume; RF, regurgitant fraction; EROA, effective regurgitant orifice area.

**Table 7.** **Key points for evaluating device stability during postoperative TEER follow-up**.

Evaluation Dimension	Key indicators	Abnormal indications
Clip position	Axial angle	Displacement/inclination
Leaflet-clip relationship	Tissue bridge width and dynamic stability	Risk of leaflet avulsion
Motion synchronization	Swing amplitude during cardiac cycle	Single leaflet device attachment (SLDA)

Major postoperative complications of TEER include inadequate attachment of single-leaflet devices, iatrogenic mitral stenosis, leaflet injury, device embolization, left ventricular outflow tract obstruction, thrombosis, and infective endocarditis. Their evaluation points are detailed in Table 8 [69].

**Table 8.** **Postoperative TEER complications and ultrasonic features**.

Complications	Diagnostic criteria	Confirmation methods
Iatrogenic mitral stenosis	MPG ≥5 mmHg Combined with clinical symptoms or MVA <1.5 cm^2^	Spectral Doppler measurement of transvalvular pressure gradient TEE 3D valve orifice area tracing
SLDA	Flail-like movement of the clip + sudden worsening of MR	Multi-plane TTE + TEE dynamic observation
Device-related thrombosis	Hypoechoic mass attached to the clip (should be differentiated from vegetations)	TEE high-frequency probe
Leaflet injury	New leaflet tear/perforation/chordae tendineae rupture	TEE 2D/3D imaging
Iatrogenic atrial septal defect (IASD)	Persistent shunt at the atrial septal puncture site (diameter >8 mm)	Color Doppler + contrast echocardiography
Infective endocarditis	Vegetations + fever + positive blood culture	Duke criteria

## 6. Conclusions

**Fig. 4.**
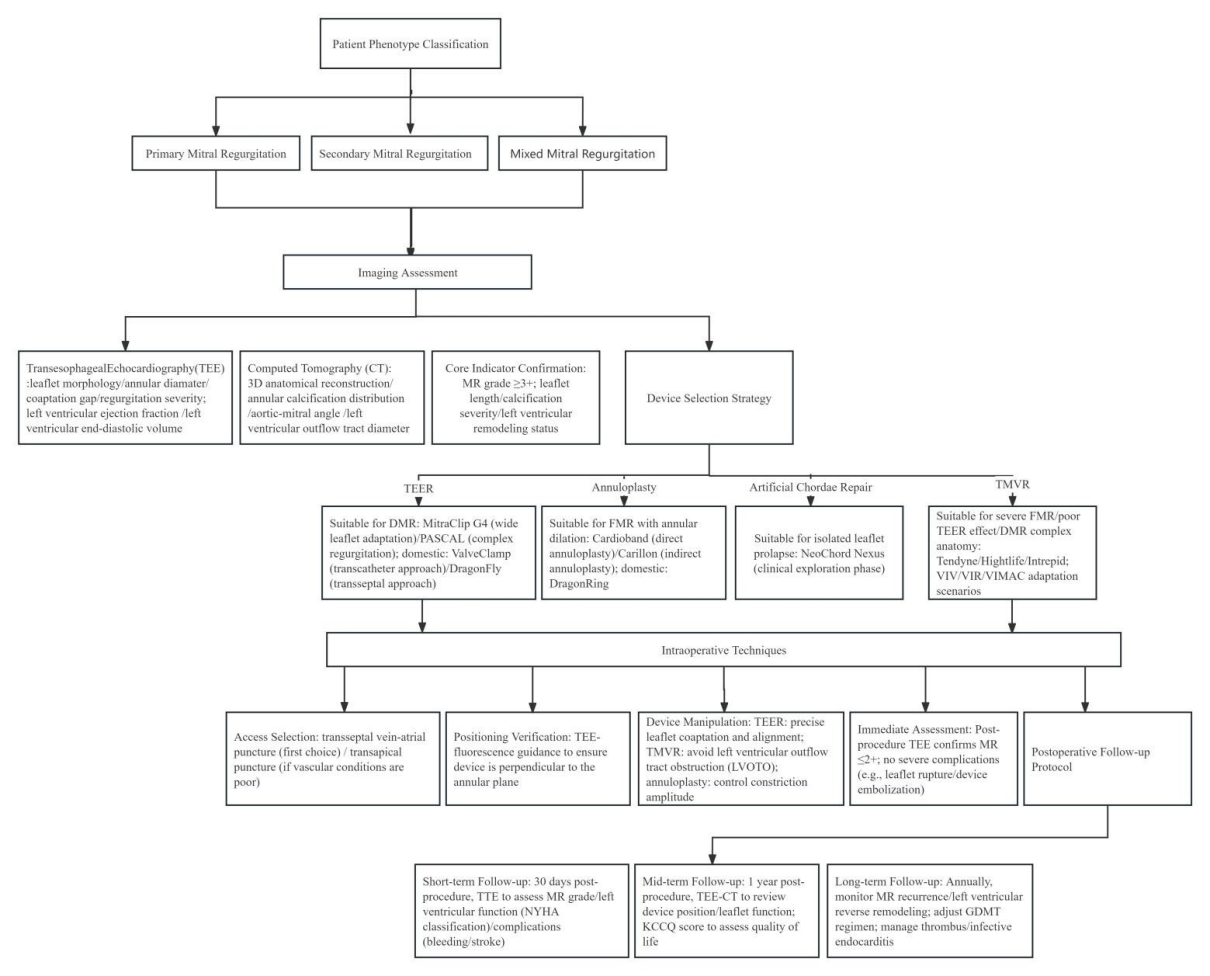
**The clinical pathway flowchart for the transcatheter intervention of mitral regurgitation (MR)**.

In recent years, interventional therapy for MR has developed rapidly with prospects for broad application. The clinical pathway flowchart for transcatheter intervention of MR, as shown in Fig. 4, covers the complete process from patient phenotype classification, key points of imaging assessment, device selection strategy, intraoperative operation techniques, and postoperative follow-up protocols. After 20 years of accumulated clinical evidence, TEER has continuously expanded its application and maintained its dominant position. The research and development of other innovative repair devices continue to evolve. A variety of TMVR systems have entered clinical trials and achieved satisfactory preliminary follow-up results. In the future, MR patients will have more minimally invasive, safer, and more effective treatment options. However, MV interventional therapy also faces many challenges, such as difficulties in positioning and fixing artificial valves, and various complications after surgery, such as left ventricular outflow tract obstruction, artificial valve thrombosis, and pericardial tamponade. Therefore, further optimization of devices, more high-level research evidence, and accumulation of experience from interventional physicians are still needed. It is believed that with the increasing improvement of MV interventional therapy, this field will continue to undergo more comprehensive development, the results of which will benefit an increasing number of MR patients.

## Abbreviations

MR, mitral regurgitation; TMVI, Transcatheter mitral valve interventions; TMVr, transcatheter mitral valve repair; TMVR, transcatheter mitral valve replacement; TEER, transcatheter edge-to-edge repair; FDA, Food and Drug Administration; NMPA, National Medical Products Administration; CE, European Conformity; MV, mitral valve; COPD, chronic obstructive pulmonary disease; COAPT, cardiovascular outcomes assessment of the MitraClip percutaneous therapy; EVEREST, Endovascular Valve Edge-to-Edge Repair Study; EXPAND G4, A Post-Market Study Assessment of the Safety and Performance of the MitraClip G4 System; DMR, degenerative mitral regurgitation; FMR, functional mitral regurgitation; AFMR, atrial functional mitral regurgitation; GDMT, guideline-directed medical therapy; LVOT, left ventricular outflow tract; MAC, mitral annular calcification; mPG, mean pressure gradient; NYHA, New York Heart Association; PISA, proximal isovelocity surface area; RF, regurgitant fraction; RVol, regurgitant volume; SLDA, single leaflet device attachment; TTE, transthoracic echocardiography; TEE, transesophageal echocardiography; M-TEER, mitral valve transcatheter edge-to-edge repair; VCW, vena contracta width; 3D, three-dimensional; VCA, vena contracta area; HFH, heart failure hospitalization; KCCQ, Kansas City Cardiomyopathy Questionnaire; STS, Society of Thoracic Surgeons; ViV, valve-in-valve; ViR, valve-in-ring; ViMAC, valve-in-calcified annulus; ESC/EACTS, European Society of Cardiology/European Association for Cardio-Thoracic Surgery; HR, Hazard Ratio; CI, Confidence Interval.
